# Etiologies of delayed diagnosis and six‐month outcome of patients with newly diagnosed advanced lung cancer with respiratory failure at initial presentation

**DOI:** 10.1111/1759-7714.13604

**Published:** 2020-08-06

**Authors:** How‐Yang Tseng, Yi‐Cheng Shen, Yen‐Sung Lin, Chih‐Yen Tu, Hung‐Jen Chen

**Affiliations:** ^1^ Division of Pulmonary and Critical Care, Department of Internal Medicine China Medical University Hospital Taichung Taiwan; ^2^ Department of Internal Medicine Tainan Municipal An‐Nan Hospital Tainan City Taiwan; ^3^ School of Medicine, College of Medicine China Medical University Taichung Taiwan

**Keywords:** Chemotherapy, intensive care unit, lung cancer, SOFA, targeted therapy

## Abstract

**Background:**

This study aimed to evaluate the characteristics of patients with newly diagnosed advanced lung cancer who initially presented with respiratory failure.

**Methods:**

This was a retrospective study which analyzed patients in the intensive care unit (ICU) with newly diagnosed advanced lung cancer who were placed on mechanical ventilation (MV). We defined newly diagnosed lung cancer as pathological or molecular results for treatment decisions not yet determined when the patient was admitted to ICU.

**Results:**

During the 14‐year inclusion period, 845 lung cancer patients requiring MV were screened. A total of 56 newly diagnosed extensive lung cancer patients were analyzed. Cancer‐related to central airway obstruction (*n* = 29, 51.8%) was the leading cause of respiratory failure. The significant etiologies of delay in the diagnosis of lung cancer were diagnostic error, mistaking cancer for tuberculosis, and missed hilar lesions. The six‐month survival rate was only 7.1% (*n* = 4). The sequential organ failure assessment (SOFA) score was significantly associated with mortality (HR = 1.142, 95% CI = 1.012–1.288, *P* = 0.031). The six‐month survival rate in patients receiving suitable targeted therapy and accepting chemotherapy and best supportive care was 40% (2/5), 0% (0/7), and 4.5% (2/44), respectively.

**Conclusions:**

Patients with newly diagnosed advanced lung cancer with acute life‐threatening respiratory failure have poor outcomes. Cancer‐related to central airway obstruction is a leading cause of respiratory failure. Diagnostic errors such as tuberculosis and missed hilar lesions are the two main etiologies of a delay in diagnosis. The SOFA score is correlated with mortality. Targeted therapy can raise the six‐month survival rates in patients with oncogenic mutation adenocarcinoma, who survive after presentation in a critical condition.

## Graphical abstract

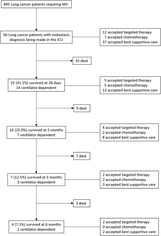

This was a retrospective study which assessed the etiologies of delayed diagnosis and characteristics of patients with newly diagnosed advanced lung cancer who initially presented with respiratory failure.

## Introduction

Lung cancer is the most frequent etiology of cancer‐related death in recent years.^1^, ^2^ In cancer patients admitted to an intensive care unit (ICU), lung cancer is also the most frequent solid cancer.^3^ The primary reason for ICU care is an acute respiratory failure.^4^, ^5^, ^6^ Results for the outcomes of lung cancer patients who need ICU care differ. One study of 49 373 lung cancer patients treated in ICU from the Surveillance, Epidemiology, and End Results (SEER)–Medicare registry showed that ICU outcomes in this study group did not change from 1992 to 2005.^5^ By contrast, outcomes for ICU treatment in lung cancer patients have demonstrated a gradually improving trend due to recent advances in critical care.^6^, ^7^ However, for lung cancer patients who develop acute respiratory failure requiring mechanical ventilation (MV), the mortality rates are reported to be around 70%.^8^, ^9^

In recent years, not all types of advanced lung cancer reveal the same poor outcomes. Targeted therapies have changed the prognosis of patients with oncogenic mutated non‐small cell lung cancer (NSCLC). Tumors that harbor epidermal growth factor receptor (*EGFR*) mutations, chromosomal rearrangements in anaplastic lymphoma kinase (*ALK*), or c‐ros oncogene 1 receptor (*ROS1*) can present dramatic responses to a targeted tyrosine kinase inhibitor (TKI),^10^, ^11^, ^12^, ^13^, ^14^ with less toxicity reported than with cytotoxic agents. Therefore, for NSCLC patients with an oncogenic mutation in ICU, the best advances in critical care with molecular targeted therapy have raised hopes of a Lazarus treatment response.^15^, ^16^, ^17^, ^18^

For patients with advanced lung cancer within one month of diagnosis, Barth *et al*. have revealed that ICU management may be helpful, and chemotherapy can improve the prognosis for patients with small cell lung cancer (SCLC).^19^ Chen *et al*. have also demonstrated that chemotherapy and targeted therapy might afford better ICU survival for treatment‐naïve, critically ill lung cancer patients.^20^

However, no study has so far focused on critical newly diagnosed lung cancer patients whose pathological or molecular results for treatment decisions have not been determined when they are admitted to ICU. In this group of ICU patients, many specific considerations deserve concern, including performing interventional procedures (pig‐tail catheter drainage, pericardiocentesis or endobronchial electrocautery), selection of tumor biopsy sites, choosing biopsy methods, oncogenic mutation determination, cancer staging, recovering multiple organ dysfunctions, and giving treatment for lung cancer. Furthermore, we should identify whether specific factors lead a patient to develop respiratory failure, even if we are unable to detect that the patient has lung cancer. With this in mind, we conducted a retrospective study to assess the etiologies of delayed diagnosis and the six‐month outcome of patients with newly diagnosed advanced lung cancer who initially presented with respiratory failure.

## Methods

### Study participants

We performed a retrospective study analyzing the outcomes of patients in the ICU with newly diagnosed advanced lung cancer placed on MV (including non‐ and invasive ventilation) because of acute life‐threatening respiratory failure between January 2006 and January 2019. Advanced lung cancer disease was defined as extensive‐stage for SCLC and stage IIIB or IV for NSCLC.^21^ Acute life‐threatening respiratory failure was defined as hypoxemia, retention of carbon dioxide, or evidence of respiratory muscle fatigue. Newly diagnosed lung cancer was defined as final pathology, *ALK* immunohistochemistry (IHC), or *EGFR* mutation results for treatment decisions not yet available when the patient was admitted to ICU.

Two electronic medical record databases from Tainan Municipal An‐Nan Hospital and China Medical University Hospital were included. Patients admitted to ICU due to postoperative care or who received treatment, ie, targeted therapy and chemotherapy, before ICU admission were excluded. The Institutional Review Board of China Medical University Hospital approved this study (CMUH 108‐REC2‐136). Informed consent was waived because of the observational and retrospective design.

We classified the main reasons for respiratory failure into direct lung cancer‐related (eg, tumor‐related central airway obstruction, diffuse pulmonary metastases, brain metastases related increased intracranial pressure, pulmonary embolism, or superior vena cava syndrome) and indirect lung cancer‐related (eg, pneumonia, cardiac pulmonary edema, severe shock, pneumonia, chronic obstructive pulmonary disease with acute exacerbation, benzodiazepine overdose) events.

### Clinical data collection, clinical assessments, and efficacy evaluations

Demographic and clinical data, including gender, age, comorbidities, smoking history, indication for MV, and do not resuscitate (DNR) orders, were collected. Comorbidities were computed with the modified Charlson Comorbidity Index.^22^ Patients who had never smoked or had smoked <100 cigarettes in their lifetime were categorized as non‐smokers.

Lung cancer‐related information, including the histological type, stage, IHC, and *EGFR* mutation status, were recorded. The pre‐ICU (within two weeks before ICU admission) Eastern Cooperative Oncology Group performance status (ECOG‐PS) was recorded.^23^

The severity of the acute illness was calculated based on data within 24 hours of MV through the sequential organ failure assessment (SOFA) ^24^ and simplified acute physiology score (SAPS) II.^25^ The management and treatment for lung cancer patients were classified as chemotherapy, targeted therapy (EGFR‐TKIs or ALK‐TKIs), and best supportive care (BSC) (without chemotherapy or targeted therapy). The adjuvant instrumental procedures to rapidly restore organ functions (eg, pig‐tail catheter drainage for pleural effusions, pericardiocentesis, or endobronchial electrocautery) were collected.

Endpoints were six‐month survival rates after the initiation of MV. Furthermore, the administration of anticancer treatments after discharge are described.

### Chest radiograph interpretation

We evaluated the series chest radiographs taken before the diagnosis of lung cancer to detect the rate of misdiagnosis. Two chest physicians, S.Y.C. and H.J.C., with more than 10 and 20 years of experience in interpretation of chest radiographs, respectively, interpreted all the posterior‐anterior chest radiographs for nodular lesions without knowledge of the clinical data. Concordance on the final results was reached after discussion. The delay was defined as the duration between the date of the pathologic diagnosis and the first radiograph on which the missed lesion could be detected according to the consensus of the two chest physicians.

### Statistical analysis

For clinical data descriptions, continuous variables are presented as median and interquartile ranges (IQRs) (25th and 75th percentiles), whereas categorical variables are expressed as percentages. Univariate and multivariate analyses for six‐month mortality were performed using the Cox proportional hazards model. Results were expressed as hazard ratios (HRs), with their 95% confidence intervals (95% CIs). The survival time after respiratory failure was estimated using the Kaplan‐Meier method, and differences among the subgroups were compared using the log‐rank test. A *P*‐value of less than 0.05 was considered statistically significant. All statistical analyses were analyzed using the SAS software package (SAS Institute, Inc., Cary, NC).

## Results

**Figure 1:**
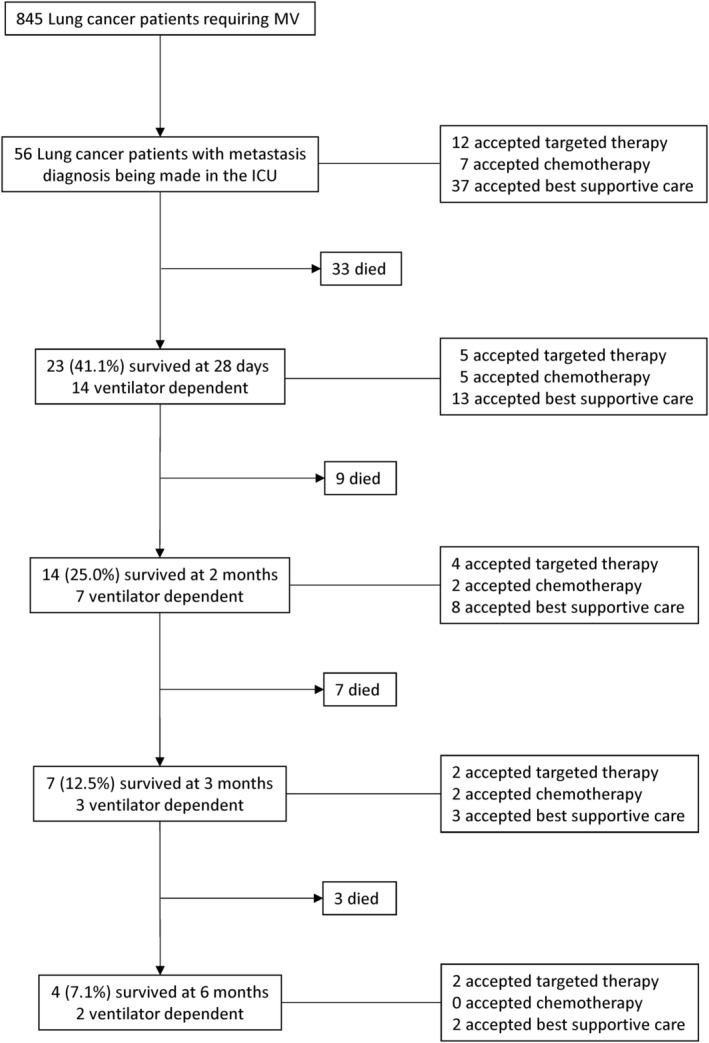
Flowchart of enrolled subjects. MV, mechanical ventilation; ICU, intensive care unit; COPD, chronic obstructive pulmonary disease.

During the 14‐year inclusion period, the medical records of 845 lung cancer patients requiring MV were initially screened. Based on the inclusion and exclusion criteria, 56 newly diagnosed extensive lung cancer patients met the criteria and were analyzed (Fig 1). Detailed baseline characteristics are summarized in Table 1. SCLC was diagnosed in six (10.7%) patients, squamous cell carcinoma was diagnosed in 16 (28.6%) patients, and adenocarcinoma was diagnosed in 29 (51.8%) patients. Out of the 29 adenocarcinoma patients, 10 had active driver mutations, distributed into *EGFR* mutation (*n* = 9), and *ALK* rearrangement (*n* = 1).

**Table 1:** Patient characteristics in the study

Characteristics	n (total = 56) (%)
Age, in years median (IQR)	70.5 (58.5–78.5)
Male gender	33 (58.9)
Smoking	33 (58.9)
Modified Charlson score, points, median (IQR)	10 (7.5–11.0)
Comorbidities	
Chronic obstructive pulmonary disease	9 (16.1)
Cardiovascular disease	26 (46.4)
Diabetes mellitus	7 (12.5)
Cirrhosis	0 (0)
Chronic kidney disease	2 (3.6)
Cerebrovascular accident	4 (7.1)
Histological type	
SCLC	6 (10.7)
Adenocarcinoma	29 (51.8)
Squamous cell carcinoma	16 (28.6)
NSCLC, NOS	2 (3.6)
Others†	3 (5.4)
IIIB–IV/extensive stage	56 (100.0)
ECOG‐PS	
Low (0–1)	53 (94.6)
High (≥2)	3 (5.4)
Respiratory failure support	
Invasive mechanical ventilation	45 (80.4)
Noninvasive mechanical ventilation	11 (19.6)
DNR	39 (69.6)
SOFA score, points, median [IQR]	6 (4.0–7.0)
SAPS II, points, median [IQR]	52 (46.0–61.0)
Anticancer therapy	
Best supportive care	37 (66.1)
Chemotherapy	7 (12.5)
Targeted therapy	12 (21.4)

† One patient had large cell carcinoma, one patient had sarcomatoid carcinoma, and one patient had adenosquamous carcinoma.

DNR, do not resuscitate; ECOG‐PS, Eastern Cooperative Oncology Group performance status; IQR, interquartile range; NOS, not otherwise specified; NSCLC, non‐small cell lung cancer; SAPS II, simplified acute physiology score, version II; SCLC, small cell lung cancer; SOFA, sequential organ failure assessment.

The reasons for respiratory failure are listed in Table 2. Direct‐lung cancer‐related complications were the main reasons for respiratory failure (43/56, 76.8%), including tumor‐related critical obstruction airway (*n* = 29, 29/56, 51.8%), diffuse lung metastases (*n* = 5), brain metastases related increased intracranial pressure (n = 5), cardiac tamponade *(n* = 2), pulmonary embolism (*n* = 1) and superior vena cava syndrome (*n* = 1). The other 13 (23.2%) patients were due to indirect‐lung cancer‐related events. Of these, pneumonia (*n* = 7) was the leading mechanism of respiratory failure.

**Table 2:** Major indication for mechanical ventilator

Characteristics	n (total = 56) (%)
Direct lung cancer related events	43 (76.8)
Central airway obstruction	29
Diffuse lung metastasis	5
Brain metastasis	5
Cardiac tamponade	2
Superior vena cava syndrome	1
Pulmonary embolism	1
Indirect lung cancer related events	13 (23.2)
Pneumonia	7
Pulmonary edema	3
Septic shock	1
COPD with acute exacerbation	1
Drug overdose	1

COPD, chronic obstructive pulmonary disease.

**Figure 2:**
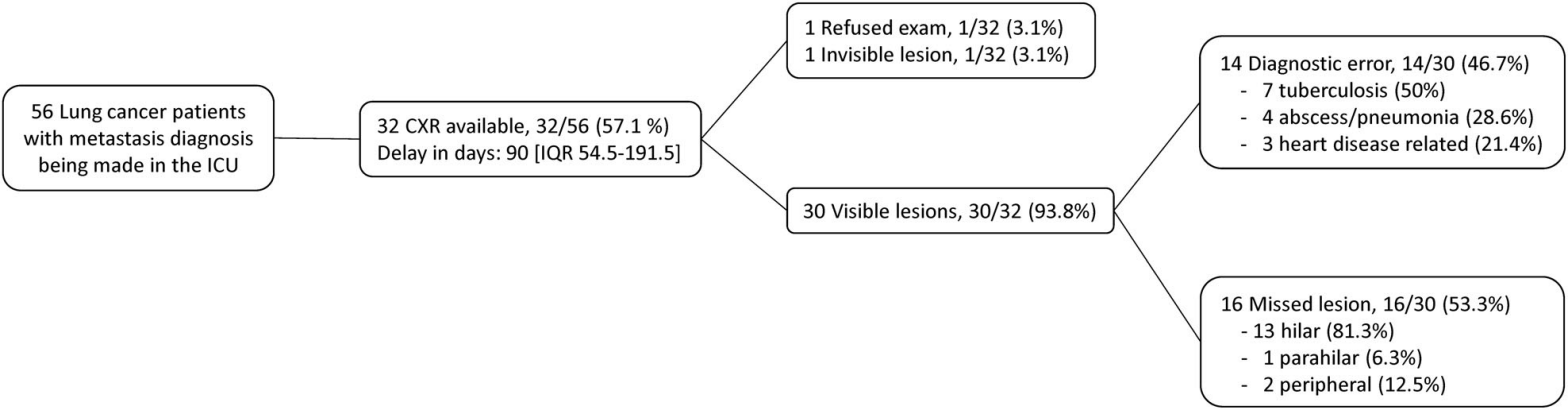
Flowchart of a previous chest radiograph is available to analyze the etiologies of delay in the diagnosis of lung cancer. CXR, chest X‐ray; ICU, intensive care unit; IQR, interquartile range.

In 32 of the 56 cases, a previous chest radiograph was available to analyze the etiologies of a delayed diagnosis (Fig 2). The median delay was 90 (54.5–191.5) days. Among the 32 patients, one patient refused to accept further examination. In one patient, no chest abnormalities could be discovered via the retrospective observation of previous radiological examinations. Of the remaining 30 patients, abnormalities could be initially detected in 14 (46.7%). However, the diagnostic error was made by the attending physician. Of these 14 cases, in seven (7/14, 50%), the lesion was misidentified as tuberculosis, in four (4/14, 28.6%) cases lung abscess/pneumonia was diagnosed, and in three (3/14, 21.4%) cases, the lesions were considered to be cardiogenic pulmonary edema or left atrial enlargement. The others (*n* = 16, 16/30, 53.3%) were missed lesions. Eight patients (8/16, 50%) were heavy smokers. Most of the lesions were located in the hilar of the lung (*n* = 13, 13/16, 81.3%).

In the 56 newly diagnosed lung cancer patients with respiratory failure, an invasive biopsy was performed during ICU stay for 53.6% (*n* = 30) of patients. There were 22 patients (39.3%) who required adjuvant procedures. Among them, seven required pleural draining, four required pericardiocentesis, and 11 required electrocautery to the tumor‐related critical airway. For acute respiratory failure, 45 (80.4%) patients received invasive MV, and 11 (19.6%) patients received noninvasive MV (Table 1). A total of 37 (66.1%) patients received BSC, seven (12.5%) patients received chemotherapy, and 12 (21.4%) received targeted therapy during their ICU stay. No patients with SCLC received chemotherapy.

Of the 12 patients who accepted rescue targeted therapy, five with oncogenic mutations received suitable TKIs (three *EGFR* L858R with erlotinib, one *EGFR* exon 19 deletion with gefitinib and one *ALK* rearrangement with ceritinib), and one with *EGFR* mutation accepted unsuitable EGFR TKI (de novo T790M with erlotinib). The remainder were either wild‐type (*n* = 5) or had an unknown (*n* = 1) mutation status. However, four patients with *EGFR* mutation adenocarcinoma did not receive the targeted therapy, including one patient who refused, while the other three had died before the molecular report was available.

The all‐cause mortality rate at day 28 after the initiation of MV was 58.9%. For those 23 patients surviving within 28 days, 14 (60.9%) could not be weaned from the MV. Five patients received chemotherapy therapy, five patients received targeted therapy (gefitinib or erlotinib), and 13 patients received BSC. Three‐ and six‐month mortality rates were 87.5 and 92.9%, respectively. Outcomes of the enrolled patients are presented in Fig 1.

Variables found associated with a *P*‐value of less than 0.25 in univariate analysis, and those considered clinically relevant (smoking and targeted therapy), were entered into a multivariate regression stepwise selection analysis in order to identify the factors independently associated with six‐month mortality. The mortality rates showed no significant differences for smoking, histological type, performance status, indication for MV, adjuvant treatment, DNR orders, and urgent anticancer chemotherapy or targeted therapy. Although mortality rates were associated with noninvasive MV in univariate analysis (unadjusted HR = 3.446, *P* = 0.001), this was not significant in the adjusted model (*P* = 0.664). After adjusting for clinical factors, the multivariate analysis showed that the SOFA score was significantly associated with six‐months mortality (HR = 1.142, 95% CI = 1.012–1.288, *P* = 0.031) (Table 3).

**Table 3:** Multivariate analysis of clinical factors associated with six‐month mortality

		Univariable analysis		Multivariable analysis		
Clinical factors	No. of patients	Hazard Ratio	*P*‐value	Hazard Ratio	95% CI	*P‐*value
Smoking						
No	23	Ref		Ref		
Yes	33	1.337	0.308	1.556	0.804–3.013	0.190
Histological type						
SCLC	06	Ref				
No/unknown driver mutation NSCLC	39	1.229	0.668			
Driver mutation NSCLC	10	0.817	0.724			
Others	01	1.120	0.919			
ECOG‐PS						
Low (0–1)	53	Ref		Ref		
High (≥ 2)	03	0.374	0.175	0.575	0.134–2.468	0.457
Severity score						
SOFA score, points, median (IQR)		1.096	0.099	1.142	1.012–1.288	0.031
SAPS II, points, median (IQR)		1.006	0.681			
Indication for mechanical ventilation						
Indirect cancer related	13	Ref				
Direct cancer related	43	0.830	0.574			
Respiratory failure support						
Invasive mechanical ventilation	45	Ref		Ref		
Noninvasive mechanical ventilation	11	3.446	0.001	1.569	0.205–11.981	0.664
Pig‐tail catheter/pericardiocentesis						
No	45	Ref				
Yes	11	0.706	0.344			
Electrocautery						
No	45	Ref				
Yes	11	0.799	0.527			
DNR						
No	17	Ref				
Yes	39	1.068	0.830			
Chemotherapy						
No	49	Ref				
Yes	07	0.729	0.439			
Targeted therapy						
No	44	Ref		Ref		
Yes	12	0.827	0.591	0.601	0.246–1.466	0.263

CI, confidence interval; DNR, do not resuscitate; ECOG‐PS, Eastern Cooperative Oncology Group performance status; IQR, interquartile range; NSCLC, non‐small cell lung cancer; Ref, reference; SAPS II, simplified acute physiology score, version II; SCLC, small cell lung cancer; SOFA, sequential organ failure assessment.

**Figure 3:**
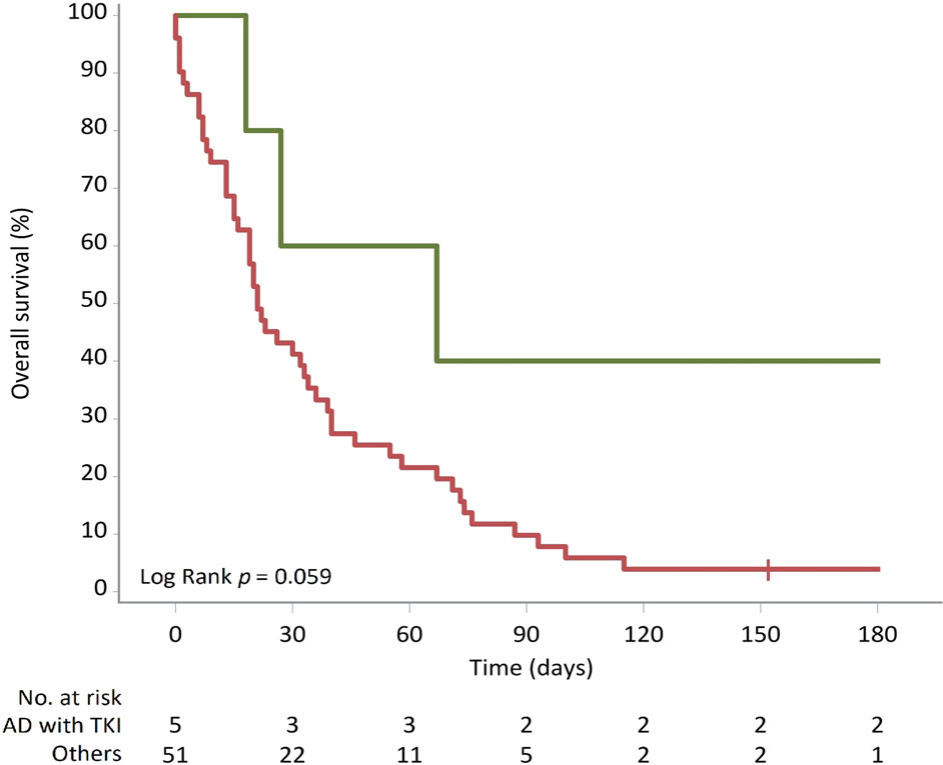
Kaplan–Meier survival curve according to the cancer treatment. 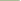 AD with TKI, patients receiving suitable targeted therapy for oncogenic mutation lung adenocarcinoma; 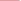 Others, the other lung cancer patients.

As shown in Table 3, reducing mortality with rescue targeted therapy is questionable. However, of the 12 patients accepting rescue targeted therapy, only five with oncogenic mutations received suitable TKIs. We further elucidate the survival impact of suitable targeted therapy for patients with oncogenic mutation lung adenocarcinoma. The six‐month survival rate in patients receiving suitable targeted therapy and accepting chemotherapy and BSC was 40% (2/5), 0% (0/7), and 4.5% (2/44), respectively. Kaplan‐Meier analysis (Fig 3) also indicated a trend towards better survival in patients receiving suitable targeted therapy (log‐rank test: *P* = 0.059).

## Discussion

This study is the first to focus on the etiologies of delayed diagnosis and the six‐month outcome of patients with newly diagnosed advanced lung cancer with acute life‐threatening respiratory failure. Cancer related to central airway obstruction (*n* = 29, 51.8%) was the leading cause of respiratory failure. The significant etiologies of delay in the diagnosis of lung cancer were diagnostic error, mistaking cancer for tuberculosis, and missed hilar lesions. A total of 23 (41.1%) patients survived at 28 days, 14 of whom (14/23, 60.9%) were ventilator dependent. The six‐month survival was only 7.1% (*n* = 4) and was associated with the SOFA score. Patients with oncogenic mutation adenocarcinoma may benefit from targeted therapies after they survive being in a critical condition.

Song *et al*. have demonstrated that the number of advanced lung cancer patients admitted to the ICU has increased over time.^7^ Zerbib *et al*. have shown that urgent chemotherapy can benefit patients with life‐threatening complications related to solid neoplasms.^26^ For treatment‐naïve, critically ill lung cancer patients, chemotherapy and targeted therapy may improve ICU survival.^19^, ^20^

However, patients with newly diagnosed advanced lung cancer with acute life‐threatening respiratory failure can be considered a distinct group. We defined newly diagnosed lung cancer as pathological or molecular results for treatment decisions not being available when the patient was admitted to ICU. Therefore, patients cannot be given precise treatment immediately. For direct lung cancer‐related respiratory failure, patients may receive invasive diagnostic workup. For indirect lung cancer‐related respiratory failure, ICU physicians must recover the organ dysfunctions related to infection, cardiogenic pulmonary edema, or acute exacerbations of chronic obstructive pulmonary disease. Unpredicted respiratory failure is another factor associated with poor prognosis in lung cancer patients, especially in those with no definite diagnosis. Nevertheless, no study has focused on the etiologies of delayed diagnosis and the natural courses of patients with newly diagnosed advanced lung cancer requiring MV support. To address this gap, our research provides informative data.

Our results appear quite different from recent studies on critically ill lung cancer patients.^19^, ^20^, ^26^ As reported, patients with SCLC have better short‐term survival than patients with other types of lung cancer.^19^, ^26^ In our study, SCLC patients were not associated with mortality because no SCLC patients had good enough activity to receive chemotherapy in ICU. Furthermore, among these advanced lung cancer patients, the cancer‐related critical obstruction airway was the leading etiology (51.8%) of respiratory failure with 11 patients requiring endobronchial electrocautery.

Various factors are associated with mortality for lung cancer patients with respiratory failure.^6^, ^8^, ^27^ We found that the six‐month mortality rates showed no significant differences for smoking, performance status, indication for MV, adjuvant treatment, DNR orders, and urgent anticancer chemotherapy or rescue targeted therapy. However, the acute severity score (SOFA) is associated with the six‐month survival rate (Table 3). This result echoes those of previous reports.^6^, ^8^, ^27^

Hsia *et al*. ^27^ and Toffart *et al*. have reported that targeted therapy cannot reduce early death (≦30 days) mortality.^18^ Our study also found that rescue targeted therapy in ICU had no survival benefits (Table 3). This result probably occurred because (i) the severity of the disease is the primary determinant of prognosis in critically ill lung cancer patients, not cancer itself; and (ii) of these 12 patients accepting rescue targeted therapy, five without oncogenic mutation and one unknown mutation patient received EGFR‐TKIs. Attending physicians adopted the strategy of “shoot first, ask later”^17^, ^28^ because they expected that EGFR‐TKIs would benefit the status of adenocarcinoma, non‐smokers, and East Asian patients.^29^

Beyond the critical management with the short‐term aim of ICU discharge, patients receiving suitable targeted therapy for oncogenic mutation lung adenocarcinoma have a better survival trend (Fig 3). Of these five patients, 40% survived over six months. Our results appear similar to those of a French study led by Toffart *et al*. who compared 14 NSCLC treatment naïve patients with oncogenic mutations in ICUs to a nonmutated group and found that the presence of oncogenic mutations was associated with improved late survival.^18^ Undoubtedly, targeted therapy in patients requiring MV support and ICU care with targetable oncogenic mutated adenocarcinoma enabled a Lazarus response and prolonged overall survival when patients can overcome the crisis of organ dysfunction.

However, the majority of survivors died within six months (*n* = 52, 92.9%). Of these four survivors, two patients were ventilator‐dependent (Fig 1). These results raise the question of how to prevent the failure to find lung cancer in patients until they develop respiratory failure. In our cohort, the 90‐day delayed diagnosis may not have had an influence on the stage of lung cancer, but it definitely had an impact on the incidence of respiratory failure. The diagnostic error was 46.7%. Misidentifications as tuberculosis and pneumonia were the leading etiologies. Agrawal *et al*. reported that out of 195 patients diagnosed with lung cancer, 79 (40%) were taking drugs for tuberculosis for at least one month due to the lack of awareness.^30^ Lung cancer presenting as lobar or segmental infiltrates has been reported in up to 11% of patients with delayed resolution of consolidations.^31^ A principal concern for physicians in the timing of evaluation of delayed resolution of consolidations may be underlying lung cancer.^32^ Missed lesions (53.3%) are the other etiology of delayed diagnosis of lung cancer. Overlying anatomical structures, such as hilum, mediastinum, ribs, blood vessels, or the heart, are far more critical for missing a nodule on chest radiograph.^33^, ^34^ In this study, 81.3% of missed lesions were located in the hilum of the lung. Cigarette smoking‐induced central airway lung cancer overlying hilar structures is a crucial factor in a missed diagnosis, and can explain why 51.8% of patients (*n* = 29) had cancer‐related central airway obstruction. The cancer‐related critical airway can also explain why lung cancer cannot be determined in patients until respiratory failure occurs.

There are some limitations in this study which must be acknowledged. First, the study design was retrospective. Second, 24 patients transferred from clinics or hospitals had no previous images available for analysis of the etiologies of delayed diagnosis. Third, the sample size was relatively small.

In conclusion, based on our results, we found that patients with newly diagnosed advanced lung cancer with acute life‐threatening respiratory failure had poor outcomes. Cancer‐related to central airway obstruction was a leading cause of respiratory failure. Diagnostic errors such as tuberculosis and missed hilar lesions were the two main etiologies of delay in diagnosis. The SOFA score was correlated with mortality, and targeted therapy could raise the six‐month survival rates of patients with oncogenic mutation adenocarcinoma, who survive after being in a critical condition.

## Disclosure

No conflicts exist for the specified authors.
